# Post-exposure prophylaxis during pandemic outbreaks

**DOI:** 10.1186/1741-7015-7-73

**Published:** 2009-12-02

**Authors:** Seyed M Moghadas, Christopher S Bowman, Gergely Röst, David N Fisman, Jianhong Wu

**Affiliations:** 1 Institute for Biodiagnostics, National Research Council Canada, Winnipeg, Manitoba, Canada; 2 Department of Mathematics and Statistics, The University of Winnipeg, Winnipeg, Manitoba, Canada; 3 Analysis and Stochastics Research Group, Hungarian Academy of Sciences, Bolyai Institute, University of Szeged, Szeged, Hungary; 4 Dalla Lana School of Public Health, University of Toronto and Ontario Agency for Health Protection and Promotion, Toronto, Ontario, Canada; 5 Centre for Disease Modelling, York Institute of Health Research, York University, Toronto, Ontario, Canada

## Abstract

**Background:**

With the rise of the second pandemic wave of the novel influenza A (H1N1) virus in the current season in the Northern Hemisphere, pandemic plans are being carefully re-evaluated, particularly for the strategic use of antiviral drugs. The recent emergence of oseltamivir-resistant in treated H1N1 patients has raised concerns about the prudent use of neuraminidase inhibitors for both treatment of ill individuals and post-exposure prophylaxis of close contacts.

**Methods:**

We extended an established population dynamical model of pandemic influenza with treatment to include post-exposure prophylaxis of close contacts. Using parameter estimates published in the literature, we simulated the model to evaluate the combined effect of treatment and prophylaxis in minimizing morbidity and mortality of pandemic infections in the context of transmissible drug resistance.

**Results:**

We demonstrated that, when transmissible resistant strains are present, post-exposure prophylaxis can promote the spread of resistance, especially when combined with aggressive treatment. For a given treatment level, there is an optimal coverage of prophylaxis that minimizes the total number of infections (final size) and this coverage decreases as a higher proportion of infected individuals are treated. We found that, when treatment is maintained at intermediate levels, limited post-exposure prophylaxis provides an optimal strategy for reducing the final size of the pandemic while minimizing the total number of deaths. We tested our results by performing a sensitivity analysis over a range of key model parameters and observed that the incidence of infection depends strongly on the transmission fitness of resistant strains.

**Conclusion:**

Our findings suggest that, in the presence of transmissible drug resistance, strategies that prioritize the treatment of only ill individuals, rather than the prophylaxis of those suspected of being exposed, are most effective in reducing the morbidity and mortality of the pandemic. The impact of post-exposure prophylaxis depends critically on the treatment level and the transmissibility of resistant strains and, therefore, enhanced surveillance and clinical monitoring for resistant mutants constitutes a key component of any comprehensive plan for antiviral drug use during an influenza pandemic.

## Background

A novel influenza A virus H1N1 has spread worldwide since its initial emergence in North America, causing the first influenza pandemic of the 21st century [1]. Public health responses to outbreaks of this nascent virus have included antiviral treatment and the isolation of infected individuals, quarantine of suspected cases and school closures as measures for the reduction of disease transmission in the population. While pandemic vaccines are currently being deployed, the timeliness of vaccine availability, limited acceptability of vaccination to groups targeted for vaccination and the limitations in vaccine supply could all increase dependence on antiviral drugs for pandemic mitigation.

Most pandemic plans support the treatment of ill individuals upon diagnosis as an efficient approach to the use of drug stockpiles [2]. However, the potential role of antiviral prophylaxis for asymptomatic individuals exposed to infectious cases remains contentious. The use of antiviral prophylaxis poses both logistical challenges (for example, due to limited drug supplies and competing distribution priorities) and could carry adverse epidemiological consequences (for example, by promoting drug resistance spread) [3,4]. In the absence of transmissible drug-resistant viral strains, models suggest that widespread use of post-exposure prophylaxis could contain influenza epidemics, particularly if applied at the outset [5,6]. However, the predictions of these models depend strongly on: the specific location of an initial outbreak; patterns of exposure to infection in localities; speed at which infected cases are diagnosed and treated; and how quickly their contacts are offered prophylaxis. Furthermore, the evolutionary responses of the virus with the generation of transmissible drug resistant mutants have been discounted in their assessment of mitigation strategies.

In the presence of drug resistance, the advisability of a post-exposure prophylaxis strategy remains uncertain. To evaluate the merits of such strategy for pandemic mitigation, we extended a previous modelling framework [7] in order to combine the effect of treatment and post-exposure prophylaxis of close contacts. Through this evaluation, we identify critical factors that influence the epidemiological outcome of these antiviral measures. In what follows, we describe the model and its parameters with their estimates, present the results of model simulations and discuss our findings and their implications for preparedness strategies in the context of the 2009 H1N1 pandemic. Details of the model structure and its analysis are provided in Additional File 1.

## Methods

We extended a previously established population dynamical model of influenza transmission with treatment of clinical infections to include post-exposure prophylaxis of close contacts of index cases [7]. We included two competing viral strains in the virus in the model - one being sensitive and the other resistant to antiviral drugs - with the assumption (consistent with empirical observations in the 2009 pandemic) that the drug-sensitive strain initially triggered the epidemic [8]. Resistance emerges and spreads in the population during the epidemic under the pressure of drug treatment.

### Model development

**Figure 1:**
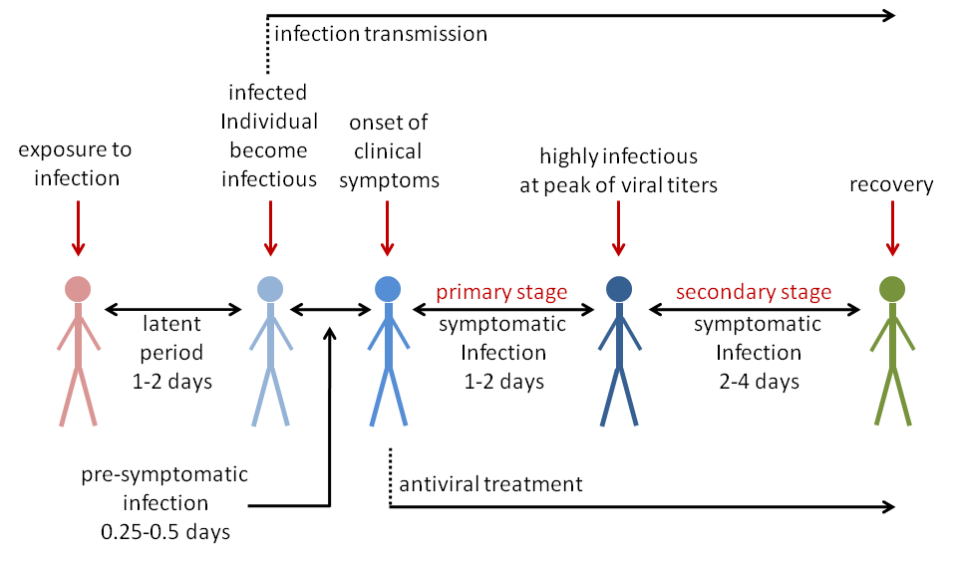
**Typical course of influenza infection leading to clinical disease with antiviral treatment**.

A typical course of symptomatic influenza infection includes an incubation period (a period during which an infected individual is asymptomatic) and clinical disease (Figure 1). We considered the incubation period as a combination of two stages: (i) a latent stage representing the interval between exposure and infectiousness in an individual; and (ii) an asymptomatic infectious stage, which represents the interval between the end of latency and the onset of clinical symptoms (referred to in this study as pre-symptomatic infection). Treatment of clinical infections is most effective when initiated within 48 h of symptom onset and we, therefore, considered two stages of symptomatic infection: (i) primary treatment that occurs during this initial window; and (ii) secondary treatment which is initiated after this window has closed. We assumed that treatment will be made available to individuals who are identified within the primary stage of symptomatic infection (Figure 1).

An important feature of influenza virus infection is its potential for transmission by infectious individuals who are truly asymptomatic or who experience sufficiently mild symptoms that they remain undiagnosed and untreated [9]. We included such individuals in our model which could, of course, mistakenly receive 'prophylaxis' (unwitting treatment) to prevent acquisition (or development) of infection. We assumed that treatment of infected individuals (symptomatic or asymptomatic) could result in selective pressure under which transmissible, antiviral-resistant strains might arise with some probability in treated patients. We assumed that an antiviral therapy would be ineffective against resistant infection [10,11].

For post-exposure prophylaxis, we targeted only close contacts of treated index cases who could be identified and prophylaxed within 24 h of case identification [12] and before the onset of symptoms. Contact tracing is always challenging for medical and public health workers [13], but we assumed that the time lag between diagnosis of clinical cases and identification of their close contacts (including households, neighbourhood clusters, school groups and workplaces) could be relatively short and, therefore, that prophylaxis would be provided prior to the onset of symptoms in individuals already infected by the index case. Further details of the model structure are provided in Additional File 1.

### Model parameters and estimates

Despite ongoing surveillance and epidemiological studies of the novel H1N1 virus, there remains substantial uncertainty in several key characteristics of the virus, such as attack rates of H1N1 illness, case-fatality rates, sensitivity of the virus to antiviral drugs and the effectiveness of treatment in reducing or preventing severe morbidity and mortality. For model parameters, we therefore relied on estimates provided in the literature for influenza A infection in humans without pre-existing immune responses. We assumed a mean latent period of 1.25 days for the disease within the estimated range 1.48 ± 0.47 days [6,14]. The probability of developing clinical symptoms is taken to be 0.6 which lies in the range 0.5 - 0.7 utilized in previous studies [5,6]. We assigned a pre-symptomatic infectious period of 0.25 day, consistent with other investigations [6,10]. We assumed a window of opportunity of 2 days (after latency) for the initiation of an effective course of antiviral treatment and a mean duration of 2.85 days for the secondary stage of symptomatic infection (during which time initiating antiviral treatment would be unhelpful) [7,10]. The mean duration of asymptomatic infection was assumed to be 4.1 days [5] and antiviral treatment was assumed to reduce the infectiousness by 60% from when treatment is initiated [4,6]. In order to produce a profile of viral shedding over time, Ferguson *et al*. [6] fitted a log-normal model to household longitudinal data on influenza transmission [15]. We used this profile to estimate the relative infectiousness in different stages of clinical infection by considering a step-function superposed on the log-normal curve with respect to transmission rates of the pre-symptomatic, primary and secondary stages of symptomatic infection (Table S1 in Additional File 1). Asymptomatic infection was assumed to be 50% less infectious than the symptomatic phase [5]. Post-exposure prophylaxis was assumed to reduce the probability of developing clinical disease by 70% and infectiousness by 80% [16] and we assumed that the probability of *de novo *emergence of resistance on treatment to be 0.018 per day during primary infection and 0.036 per day during secondary infection, with a range of resistance rates extending as high as 0.072 per day [4,10]. In our model, these rates correspond to estimates provided in oseltamivir clinical trials, during which 1% - 4% of treated adults [17] and 5% - 6% of treated children were found to shed resistant viruses [18], although more recent studies have reported resistance in 16% - 18% of oseltamivir-treated children [19,20]. Resistance rates were reduced by 80% for individuals who developed symptomatic infection on prophylaxis [16]. Finally, we assumed that the resistant strain would sustain a 'fitness cost', such that the baseline transmissibility of the resistant strain was 90% of that seen with the non-resistant strain. Both treatment and prophylaxis were considered to be ineffective against infections caused by exposure to resistant viruses [21,22].

To estimate the baseline transmission rate of the drug sensitive strain, we used the final size equation and the expression for the basic reproduction number of disease [23], defined as the average number of new infections generated by a single infected case introduced into an entirely susceptible population (see Additional File 1). The reproduction number of the sensitive strain in the absence of antiviral therapy is given by

In this expression: *β *is the transmission rate of the sensitive strain; *S*_0 _is the initial size of the susceptible population, *p* is the probability of developing clinical disease; *δ*_*A*_, *δ*_*P *_and *δ*_*U *_are the relative transmissibility of the virus during asymptomatic, pre-symptomatic and secondary stage of symptomatic infection, respectively; *τ *and *n *represent the durations of pre-symptomatic and primary stage of symptomatic infection; and *μ*_*A *_and *μ*_*U *_are the mean infectious periods of asymptomatic and secondary stage of symptomatic infection. Given the range 25% - 50% of clinical attack rate of a pandemic strain [24], the reproduction number  varies between 1.25 and 1.85. With 90% fitness of resistance (as the baseline value for simulations), the reproduction number of the resistant strain () varies in the range 1.13 - 1.67. Epidemiological analyses using data collected during the early stages of the current H1N1 pandemic suggest that the reproduction number of this new virus varies in the range 1.3 - 1.7 [25,26]. We therefore used  = 1.6 as the baseline value for our simulations, which is slightly lower than estimates for the 1918 pandemic [27] and lies within the range estimated for the 1957 pandemic [24]. When treatment and post-exposure prophylaxis are applied, the number of new infections generated by a single infected case is determined by the control reproduction number of disease (*R*_*c*_), as detailed in Additional File 1. The principal aim of public health measures is to bring *R*_*c *_below one, so that the spread of disease is contained.

## Results

**Figure 2:**
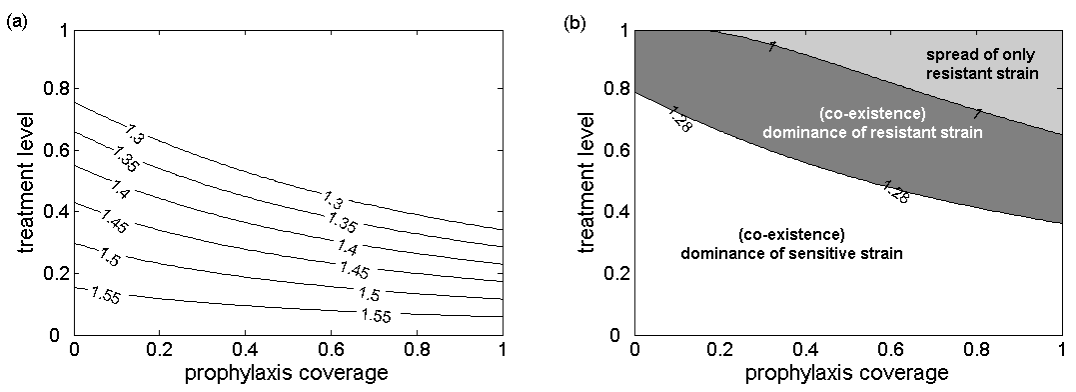
**Effect of treatment and prophylaxis on the reproduction number and spread of sensitive and resistant viruses**. (a) The combined effect of treatment and prophylaxis in reducing the control reproduction number of disease (*R*_*c*_) with  = 1.6. (b) The region for co-existence and dominance of sensitive and resistant strains as a function of treatment level of indexed cases and prophylaxis coverage of close contacts with  = 1.6. The dominance of the sensitive (resistant) strain in white region (in the grey area) corresponds to the greater reproduction number of that strain; in the light grey area, the reproduction number of the sensitive strain is less than one (that is, the sensitive infection is contained) and only outbreaks of the resistant strain may occur. Baseline values of other parameters are given in Table S1 of Additional File 1.

In order to realize the model, we considered a population of *S*_0 _= 100,000 individuals, with the introduction of an infected (drug-sensitive) case at time *t *= 0, and assumed that the treatment of clinical cases began one day after the onset of symptoms. For the baseline value of  = 1.6( = 1.44), Figure 2a shows contour plots for the control reproduction number *R*_*c *_as a function of treatment level of indexed cases and prophylaxis coverage of close contacts (the proportion of close contacts that are given prophylaxis). As the treatment level increases, the effect of post-exposure prophylaxis becomes more pronounced in decreasing *R*_*c*_, but also makes a greater contribution to the spread of resistance due to substantial reduction in the transmission of drug sensitive strain. Figure 2b illustrates the regions for: (i) co-existence of sensitive and resistant infections (white area) where sensitive strain dominates; (ii) co-existence of sensitive and resistant infections (dark grey area) where resistant strain dominates; and (iii) spread of only resistant strain (light grey area) where drug sensitive infection is contained. For a sufficiently low treatment level (below approximately 40%) the resistant strain may be out-competed by the dominant drug-sensitive competitor and large outbreaks of sensitive infections can occur. When the treatment level increases above 40%, the coverage of post-exposure prophylaxis is a key parameter determining the outcome of competitive interference between different strains: higher prophylaxis coverage limits the spread of the sensitive strain, thereby shifting the competitive balance in favour of the resistant strain (grey area).

**Figure 3:**
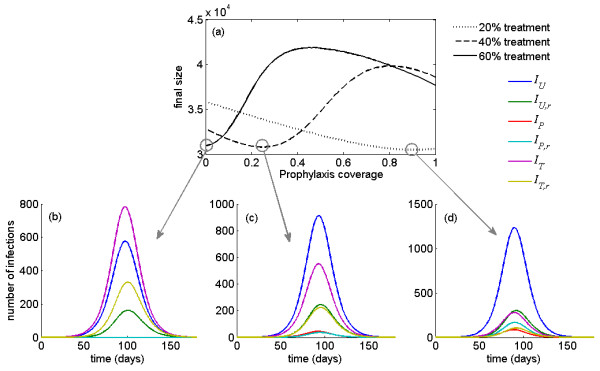
**Optimal prophylaxis coverage for various treatment levels with the corresponding time-courses of epidemic**. (a) Total number of infections (final size) as a function of prophylaxis coverage of close contacts for different treatment levels with  = 1.6. For the minimum final size corresponding to each treatment level (circled in (a)), the time courses of disease outbreaks are illustrated in (b), (c) and (d) for both sensitive and resistant infections. Each curve in the lower panel of the figure represents an epidemic curve for a given treatment group (*U *= untreated, *P *= propylaxed, *T *= treated), with subscript *r *denoting whether or not the virus is resistant to antiviral drugs.

To illustrate the effect of post-exposure prophylaxis on the epidemic size, we performed simulations for different treatment levels and calculated the final size of infection by varying prophylaxis coverage. Figure 3a shows that, as the treatment level increases, prophylaxis coverage should be reduced to minimize the total number of infections. This is illustrated for the 20%, 40% and 60% treatment levels (corresponding to dotted, dashed and solid lines), in which the minimum in the final size occurs, respectively, at 90%, 26% and 0% prophylaxis coverage of close contacts. The time courses of disease epidemics corresponding to the minimum final size (circled in Figure 3a) are shown in Figures 3b, c and 3d, which indicate no significant differences in the peak times of outbreaks, although the relative proportion of virus types (sensitive or resistant) do change.

**Figure 4:**
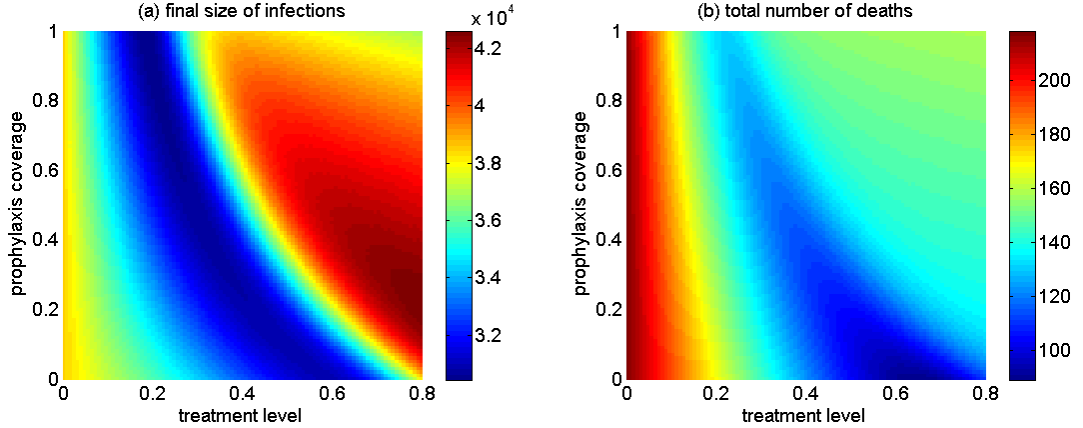
**The combined effect of treatment level of indexed cases and prophylaxis coverage of close contacts**. (a) The total number of infections (final size); (b) the total number of deaths, with  = 1.6.

We ran further simulations in order to explore the combined effects of treatment and post-exposure prophylaxis on the final epidemic sizes, and total deaths. Figure 4a shows that, in the presence of drug-resistant strains, final size is minimized at intermediate levels of treatment (represented by the dark blue valley) and requires a steep reduction in prophylaxis coverage (from 90% to 0% of close contacts) as the treatment level increases (from 20% to 60%). While the total number of infections can be minimized at low treatment levels with relatively high prophylaxis coverage, our simulations suggest that high treatment levels without prophylaxis are most effective in reducing the total number of deaths (Figure 4b).

**Figure 5:**
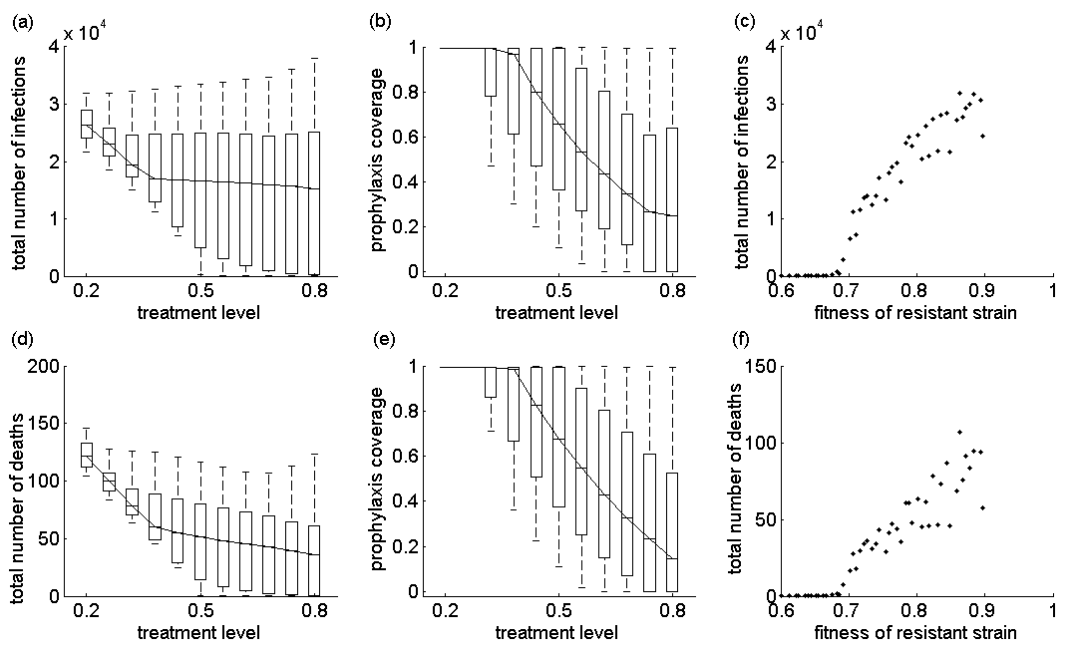
**Effect of parameter changes on the optimal prophylaxis coverage, final size and deaths**. Sensitivity analyses for the effect of treatment level on the minimum number of infections and deaths (a, d), corresponding to the optimal prophylaxis coverage as a function of treatment level (b, e). For the optimal prophylaxis coverage, (c) and (f) illustrate the sensitivity of the minimum number of infections and deaths to the transmission fitness of the resistant strain.

We tested our results for the final size of the pandemic by performing a sensitivity analysis for a range of key parameters, including: the fitness of resistant virus (*δ*_*r*_, range: 0.6 - 0.9); the probability of developing clinical disease without prophylaxis (*p*, range: 0.5 - 0.7); the probability of developing clinical disease with prophylaxis (*p*_*P*_, range: 0.2 - 0.4); the rate of developing *de novo *resistance due to treatment within primary stage of symptomatic infection (*ρ*, range: 0.009 - 0.036); the rate of developing *de novo *resistance due to treatment during secondary stage of symptomatic infection (*α*, range: 0.018- 0.072); the rate of developing *de novo *resistance due to prophylaxis within primary stage of symptomatic infection (*ρ*_*P*_, range: 0.0018 - 0.0072); and the rate of developing *de novo *resistance due to prophylaxis during secondary stage of symptomatic infection (*α*_*P*_, range: 0.0036 - 0.0144). In the sensitivity analyses, other parameters were fixed at their baseline values (Table S1 in Additional File 1), with  = 1.6. Using the Latin Hypercube Sampling technique (see Additional File 1), we simulated the model when treatment level varied in the range 20% - 80% in order to determine the optimal prophylaxis coverage that minimizes the total number of infections. Figure 5a shows box-plots for the minimum total number of infections as a function of treatment level, corresponding to box-plots illustrated in Figure 5b for the optimal prophylaxis coverage. For low treatment levels, the total number of infections is minimized with high prophylaxis coverage of close contacts (Figure 5a). However, when treatment increases to moderate and high levels (above approximately 35%), resistance spreads more readily and a reduction in prophylaxis coverage is required to achieve minimum infections (Figure 5b), which is consistent with the results shown in Figure 4a. From the sensitivity analyses for the optimal prophylaxis coverage, we made an important observation - that the minimum number of infections is strongly correlated with the fitness of resistant strain, as illustrated in Figure 5c. We obtained similar results for minimizing the mortality of the population (Figures 5d, e, f), with a more pronounced effect of the treatment in reducing mortality than morbidity at the optimal prophylaxis coverage.

## Discussion

Post-exposure prophylaxis has been considered in previous modelling studies and projected to be a useful measure for mitigating the spread of pandemic infections in the absence of antiviral resistance [5,6,28]. Stochastic models, developed to simulate potential outbreaks of the avian influenza H5N1 strain in rural Southeast Asia, predicted that a pandemic could be contained at the source through a combination of targeted blanket prophylaxis of close contacts and social distancing. In addition to being a useful measure in reducing the secondary influenza transmission in households [29,30], targeted prophylaxis has been shown to be effective in reducing the overall attack rates and in slowing disease spread in the community [28]. Furthermore, an economic evaluation of mitigation strategies suggests that post-exposure prophylaxis provides a cost-effective strategy during an influenza pandemic [16]. However, despite such potential benefits, the emergence of drug resistance poses a significant threat to the effectiveness of post-exposure prophylaxis and the use of limited drug stockpiles.

To our knowledge, this study represents the first modelling attempt to evaluate the projected effectiveness of prophylaxis of close contacts of infected individuals treated within 48 h of symptoms onset in the context of drug resistance. Our model incorporates several important parameters that impact the epidemiological outcome of any antiviral strategy, including: the population level of treatment; the rate of *de novo *resistance in treated patients; the transmission fitness of resistant strains; and the prophylaxis coverage of close contacts. When resistance is included, the projected effectiveness of prophylaxis-based strategies in our model differs from those presented in previously published papers [5,6,16,28]. The model extends our previous work on pandemic influenza with antiviral resistance [7,10,11] and illustrates optimal scenarios when treatment is combined with post-exposure prophylaxis.

Our results show that the impact of post-exposure prophylaxis depends on the treatment level and the transmissibility of resistant strains. For low treatment levels, the sensitive strain has a fitness advantage and spreads quickly, and therefore a limited number of resistant cases are generated. In this situation, post-exposure prophylaxis is projected to lower the incidence of disease in the population, consistent with findings of previous work [4,31]. However, increasing treatment to moderate and higher levels limits the incidence of drug sensitive infection and shifts the competitive balance in favour of the resistant strain. With a sufficiently high transmission fitness of the resistant strain, our simulations and sensitivity analyses demonstrate that post-exposure prophylaxis will actually enhance the spread of drug resistance. This could effectively counterbalance reductions in disease transmission and illness associated with antiviral prophylaxis and adjunctive prophylaxis would offer no advantages (and possible disadvantages) relative to an antiviral strategy that restricts provision of antiviral drugs for the timely treatment of diagnosed cases. We project that, when antiviral resistance is considered, strategies that prioritize the treatment of *ill *individuals will be most effective in reducing morbidity and mortality during an influenza pandemic.

Our results corroborate the findings of a recent assessment of the effectiveness of post-exposure prophylaxis in nursing homes [32]. If resistance developed, post-exposure prophylaxis was shown to be significantly less efficacious in protecting patients. There was more selection for resistance during continuous prophylaxis which could result in an increased prevalence of resistant viruses. Subsequent transmission of resistance to visitors and those caring for patients with influenza could lead to the spread of drug resistant viruses in the wider-community; thereby substantially limiting the impact of antiviral measures. In this context, it has been suggested that allocating different drugs for treatment and prophylaxis may constrain the development of resistance [31]. However, it is important to note that the emergence and spread of resistance is also influenced by other factors, such as compensatory mutations that ameliorate the fitness costs associated with resistance evolution. For example, a dramatic rise in the emergence of drug resistance in seasonal influenza H1N1 viruses has been reported worldwide, even in geographic areas where the drugs have been infrequently used. Recent work suggests that the globally increasing frequency of resistance is more likely attributable to its interaction with fitness-enhancing mutations rather than to direct drug selection pressure [33]. Thus, the use of drugs may not be the sole factor in the elevated population incidence of drug resistance.

This study is highly relevant to decisions currently being made by public health agencies faced with trying to control the 2009 influenza A (H1N1) pandemic. Although initial investigations documented the sensitivity of the pandemic influenza strain to neuraminidase inhibitors (that is, oseltamivir and zanamivir) [8], the recent development of drug-resistance in patients treated with oseltamivir is extremely concerning. Of 31 confirmed cases of H1N1 oseltamivir-resistance (with the same H275Y mutation) reported as of 4 October 2009, 12 were associated with post-exposure prophylaxis with this drug [34]. Oseltamivir-resistance was also isolated in a patient travelling between San Francisco and Hong Kong, with no documented treatment with oseltamivir (3 July 2009), which raises concerns about the transmissibility of resistant mutants (as well as the rapid international dissemination of such mutants). A similar case of resistance in someone who had not been previously treated with oseltamivir was identified in Japan on 22 August 2009. A recent Centres for Disease Control and Prevention report provides further evidence for the possible transmission of oseltamivir-resistant viral strains among individuals involved in a programme of mass oseltamivir prophylaxis during an outbreak of influenza-like illnesses at a summer camp [35]. Given the extensive use of drugs in some affected countries, enhancement of the prevalence of oseltamivir resistant viral strains in the current pandemic does appear likely. Therefore, enhanced surveillance and clinical monitoring for the rapid detection of resistant viruses and the determination of transmission fitness constitutes a most desirable complement to any programme involving widespread distribution of antiviral medications for pandemic mitigation.

Like any mathematical model, our model is subject to limitations, including the simplifying assumption of homogeneity in the population interactions and the absence of stochastic ('random') effects that may play a dominant role in disease spread early in an epidemic. Recent studies provide a solid foundation for the extension of our model to include heterogeneity in population dynamics of disease transmission [36,37]. In the absence of data on the novel pandemic influenza strain, we have used antiviral effectiveness estimates derived from studies performed during seasonal influenza [38] and we have used published parameters which are subject to some degree of uncertainty. Nonetheless, while these assumptions may limit the precise quantitative predictive ability of our model, we note that our projections were robust in the face of wide-ranging sensitivity analyses and we emphasize the qualitative aspects of this study which should help public health professionals as they strive to provide guidance on the best use of antiviral drugs in the face of substantial uncertainty.

## Conclusion

In the presence of transmissible drug resistant viruses, the strategic use of antiviral drugs is crucial to the mitigation of the impact of pandemic influenza on individuals and the population as a whole. Our findings suggest that strategies that prioritize the treatment of only ill individuals, rather than prophylaxis of those suspected of having been exposed, are most effective in reducing morbidity and mortality of the pandemic.

## Competing interests

The authors declare that they have no competing interests.

## Authors' contributions

SM and CB developed the model. GR and JW analysed the model theoretically. CB conceived and performed the experiments. SM, CB, GR, DF and JW contributed reagents/materials/analysis tools. SM and DF designed the study and wrote the paper. All the authors have read the final version of the paper and approved it.

## Pre-publication history

The pre-publication history for this paper can be accessed here:

http://www.biomedcentral.com/1741-7015/7/73/prepub

## Supplementary Material

Additional file 1 **Post-exposure prophylaxis during pandemic outbreaks**. Model structure and its analyses with parameter values used for simulations are provided. Click here for file
