# Supplementary material for: Post-exposure prophylaxis during pandemic outbreaks
Source: BMC Med. 2009 Dec 2;7:73. doi: 10.1186/1741-7015-7-73 (PMC2794871; doi:10.1186/1741-7015-7-73)
Supplement: Additional file 1 — Post-exposure prophylaxis during pandemic outbreaks. Model structure and its analyses with parameter values used for simulations are provided. [file 1741-7015-7-73-S1.PDF]

Supplementary Information:
Post-exposure prophylaxis during pandemic outbreaks

Seyed M. Moghadas,[1,2] Christopher S. Bowman,[1] Gergely Röst,[3] David N. Fisman,[4] Jianhong Wu[5]

November 21, 2009

[1]*Institute for Biodiagnostics, National Research Council Canada, Winnipeg, Manitoba, Canada,*
[2]*Department of Mathematics and Statistics, The University of Winnipeg, Winnipeg, Manitoba, Canada*
[3]*Analysis and Stochastics Research Group, Hungarian Academy of Sciences, Bolyai Institute, University of Szeged, Szeged, Hungary*
[4]*Dalla Lana School of Public Health, University of Toronto and Ontario Agency for Health Protection and Promotion, Toronto, Ontario, Canada*
[5]*Centre for Disease Modelling, York Institute of Health Research, York University, Toronto, Ontario, Canada*

This supplementary material describes details of the model structure and its analysis, and provides estimates of parameter values used for simulations and sensitivity analyses.

1 Model Structure

Following previous work (Alexander *et al.*, 2007a,b), we develop a model to include prophylaxis of close contacts with infected individuals in addition to the treatment of clinically diagnosed cases. For the use of antivirals to treat ill individuals, we consider several population compartments comprising susceptible (S), exposed (E, E_r), asymptomatic (A, A_r), untreated symptomatic (I_U, $I_{U,r}$), and treated symptomatic (I_T, $I_{T,r}$) individuals, where the index r is used to represent classes of individuals associated with the resistant infection. The transmission dynamics of drug sensitive and drug resistant viruses are described by

$$\begin{aligned} S'(t) &= -\beta Q(t)S(t), \\ E'(t) &= \beta Q_s(t)S(t) - \mu_E E(t), \\ E_r'(t) &= \beta Q_r(t)S(t) - \mu_E E_r(t), \end{aligned} \tag{1}$$

where β is the baseline transmission rate of the sensitive strain; $1/\mu_E$ represents the mean latent period (assumed to be the same for E and E_r classes), and βQ_s and βQ_r are components of the force of infection βQ (yet to be formulated), and comprise the infectious compartments of sensitive and resistant strains, respectively.

To formulate the dynamics of symptomatic infection after the latent period, we considered a similar structure to Alexander *et al.*, 2007b by dividing the clinical course of disease into three stages of: (i) pre-symptomatic infection, during which transmission can occur without clinical symptoms; (ii) primary stage of symptomatic infection after the onset of symptoms, which represents the window of opportunity for start of

treatment; and (iii) secondary stage of symptomatic infection following the window of opportunity. Let $r(a)$ represent the treatment rate at elapsed time a since an exposed individual becomes infectious, and $i_U(t,a)$ and $i_T(t,a)$ be respectively the densities of untreated and treated individuals infected with the sensitive strain at current time t. Let ρ be the rate at which treated individuals develop drug-resistance within the window of opportunity, and $i_{U,r}(t,a)$ and $i_{T,r}(t,a)$ denote the densities of untreated and treated individuals infected with the resistant strain, respectively. Then, the equations for the primary stage of symptomatic infection can be expressed as (Alexander *et al.*, 2007a)

$$\Big(\frac{\partial}{\partial t}+\frac{\partial}{\partial a}\Big)i_U(t,a) = -r(a)i_U(t,a), \tag{2}$$

$$\Big(\frac{\partial}{\partial t}+\frac{\partial}{\partial a}\Big)i_T(t,a) = r(a)i_U(t,a) - \rho i_T(t,a), \tag{3}$$

$$\Big(\frac{\partial}{\partial t}+\frac{\partial}{\partial a}\Big)i_{U,r}(t,a) = -r(a)i_{U,r}(t,a), \tag{4}$$

$$\Big(\frac{\partial}{\partial t}+\frac{\partial}{\partial a}\Big)i_{T,r}(t,a) = r(a)i_{U,r}(t,a) + \rho i_T(t,a), \tag{5}$$

subject to the following boundary conditions

$$i_U(t,0) = p\mu_E E(t) \qquad i_{U,r}(t,0) = p\mu_E E_r(t), \qquad i_T(t,0) = 0, \qquad i_{T,r}(t,0) = 0,$$

where p is the probability of developing clinical symptoms. Solving (2)−(5), we obtain for $t \geq a$

$$i_U(t,a) = p\mu_E E(t-a)q(a), \tag{6}$$

$$i_T(t,a) = p\mu_E E(t-a)\big(1-q(a)\big) - p\mu_E E(t-a)V(a), \tag{7}$$

$$i_{U,r}(t,a) = p\mu_E E_r(t-a)q(a), \tag{8}$$

$$i_{T,r}(t,a) = p\mu_E E_r(t-a)\big(1-q(a)\big) + p\mu_E E(t-a)V(a), \tag{9}$$

where

$$V(a) = 1 - v(a) - v(a)\rho\int_0^a \frac{q(x)}{v(x)}dx, \tag{10}$$

$$v(a) \equiv \mathrm{e}^{-\int_0^a \rho du}, \qquad q(a) = \mathrm{e}^{-\int_0^a r(u)du}, \quad 0 \leq a \leq n, \tag{11}$$

and n represents the size of the window of opportunity. Using the above notation, we define $q := q(n)$, where $1-q$ represents the fraction of clinical infections which receives treatment; the parameter $v := v(n)$ may be interpreted as the fraction of infected individuals undergoing therapy without developing drug-resistance during primary stage of symptomatic infection; and $V := V(n)$ is the fraction of treated individuals which develops drug-resistance within the window of opportunity. Thus, the dynamics of symptomatic infections for $a \geq n$ is given by

$$\begin{aligned} I'_U(t) &= i_U(t,n) - (\mu_U + d_U)I_U(t), \\ I'_T(t) &= i_T(t,n) - (\mu_T + d_T + \alpha)I_T(t), \\ I'_{U,r}(t) &= i_{U,r}(t,n) - (\mu_U + d_{U,r})I_{U,r}(t), \\ I'_{T,r}(t) &= i_{T,r}(t,n) + \alpha I_T - (\mu_U + d_{U,r})I_{T,r}(t), \end{aligned} \tag{12}$$

where μ_U and μ_T are the recovery rates of untreated and treated symptomatic infections (during secondary

stage), respectively; d_U, $d_{U,r}$ and d_T stand for disease-induced mortality rates of untreated and treated symptomatic infections, respectively; and α is the rate of developing drug-resistance during secondary stage of symptomatic infection. Hence, the dynamics of infection with antiviral treatment is governed by equations (1) and (12).

We now integrate the above model with a post-exposure prophylaxis strategy. To do so, we need to consider close contacts of infected individuals who are currently undergoing treatment or will be clinically identified at a later stage (within the window of opportunity) to receive treatment. It is assumed that contacts of those who are not clinically recognizable (e.g., asymptomatic infection) cannot be traced. With the above notation, the probability that an infected individual receives treatment within a units of time after developing clinical symptoms is given by $1-q(a)$. Since the size of the window of opportunity is greater than the latent period, post-exposure prophylaxis may be targeted to close contacts of diagnosed cases in the i_U and $i_{U,r}$ classes that have occurred during the past $1/\mu_E$ units of time, before these contacts develop symptoms. In practice, however, tracing contacts may not be instantaneously feasible with start of treatment of indexed cases. We therefore assumed that the time lag between diagnosis of clinical cases and identification of their close contacts (including households, neighborhood clusters, school groups, and workplaces) is relatively short and comparable to the average duration of pre-symptomatic stage, denoted by τ. Specifically, we assume that prophylaxis is administered to close contacts that have occurred with an infectious individual within the past $1/\mu_E-\tau$ units of time (which is one day based on parameter values given in Table 1). This means that an exposed person (indexed as a close contact) will receive prophylaxis during latency following a contact at time a, with the probability

$$\begin{aligned}\xi(a) &= \left\{\begin{matrix}\text{probability that an infected}\\ \text{individual is diagnosed}\\ \text{within } a+1/\mu_E-\tau \text{ units}\\ \text{of time during symptomatic}\\ \text{infection}\end{matrix}\right\} - \left\{\begin{matrix}\text{probability that an infected}\\ \text{individual is diagnosed}\\ \text{within } a \text{ units of time during}\\ \text{symptomatic infection}\end{matrix}\right\} = \left\{\begin{matrix}\text{probability that a close con-}\\ \text{tact of an infectious indi-}\\ \text{vidual receives prophylaxis}\\ \text{within } 1/\mu_E-\tau \text{ units of time}\\ \text{following exposure}\end{matrix}\right\}\\ &= \big[1-q(a+1/\mu_E-\tau)\big]-\big[1-q(a)\big] = q(a)-q(a+1/\mu_E-\tau).\end{aligned}$$

To express the equations for the dynamics of infectious classes with prophylaxis, we let E_P and $E_{P,r}$ denote the classes of individuals exposed to sensitive and resistant strains, respectively, who receive prophylaxis. Thus, we have

$$E_P'(t) = \beta S(t)Q_P(t)-\mu_E E_P(t), \quad (13)$$

$$E_{P,r}'(t) = \beta S(t)Q_{P,r}(t)-\mu_E E_{P,r}(t), \quad (14)$$

where Q_P and $Q_{P,r}$ denote the compartments of the force of infection associated with prophylaxis. Similar to the treatment scenario, we let $i_P(t,a)$ and $i_{P,r}(t,a)$ represent, respectively, the densities of individuals infected with sensitive and resistant strains at current time t. Assuming that infected individuals with prophylaxis develop drug-resistance during the primary stage of symptomatic infection at a constant rate ρ_P, we obtain

$$\left(\frac{\partial}{\partial t}+\frac{\partial}{\partial a}\right)i_P(t,a) = -\rho_P i_P(t,a), \quad (15)$$

$$\left(\frac{\partial}{\partial t}+\frac{\partial}{\partial a}\right)i_{P,r}(t,a) = \rho_P i_P(t,a), \quad (16)$$

subject to the following boundary conditions

$$i_P(t,0) = p_P \mu_E E_P(t), \qquad i_{P,r}(t,0) = p\mu_E E_{P,r}(t),$$

where p_P is the reduced probability of developing clinical symptoms due to prophylaxis. Solving (15)−(16), we have for $t \geq a$

$$i_P(t,a) = p_P \mu_E E_P(t-a)\mathrm{e}^{-\rho_P a}, \tag{17}$$

$$i_{P,r}(t,a) = p\mu_E E_{P,r}(t-a) + p_P \mu_E E_P(t-a)\big(1-\mathrm{e}^{-\rho_P a}\big). \tag{18}$$

Let I_P and $I_{P,r}$ represent the associated compartments of secondary stage of symptomatic infections. Then, we have for $a \geq n$

$$I_P'(t) = i_P(t,n) - (\mu_T + d_T + \alpha_P)I_P(t), \tag{19}$$

$$I_{P,r}'(t) = i_{P,r}(t,n) + \alpha_P I_P(t) - (\mu_U + d_{U,r})I_{P,r}(t), \tag{20}$$

where α_P is the rate at which infected individuals with prophylaxis develop drug-resistance during secondary stage of symptomatic infection.

Finally, denoting the corresponding classes of asymptomatic infection by A, A_r, A_P, and $A_{P,r}$, and assuming that prophylaxis has no effect in reducing the probability of developing symptoms of individuals infected with the resistant strain, we have

$$A'(t) = (1-p)\mu_E E(t) - \mu_A A, \tag{21}$$

$$A_r'(t) = (1-p)\mu_E E_r(t) - \mu_A A_r, \tag{22}$$

$$A_P'(t) = (1-p_P)\mu_E E_P(t) - \mu_{A,P} A_P, \tag{23}$$

$$A_{P,r}'(t) = (1-p)\mu_E E_{P,r}(t) - \mu_A A_{P,r}, \tag{24}$$

where $1/\mu_A$ and $1/\mu_{A,P}$ represent the mean period of asymptomatic infection without and with prophylaxis, respectively.

We are now in the position to express the force of infection in terms of its components. Let θ denote the fraction of close contacts that are given prophylaxis, referred to as prophylaxis coverage in this study. Since treatment or prophylaxis has no effect in reducing the infectiousness level of individuals infected with the resistant strain, these components are given by

a) contacts with individuals infected with the sensitive strain who are asymptomatic, or those who are symptomatic and not diagnosed/treated:

$$\begin{aligned} Q_S(t) \quad = \quad & \delta_A A(t) + \delta_P \delta_A A_P(t) + \delta_U I_U(t) + \delta_T \delta_U I_T(t) + \delta_P \delta_U I_P + (1-\theta)\delta_P \int_0^\tau \delta_S i_P(t,a)da \\ & + (1-\theta)\delta_P \int_\tau^n i_P(t,a)da + (1-\theta)\delta_T \int_\tau^n i_T(t,a)da \\ & + \delta_S \int_0^\tau \big(1-\theta\xi(a)\big) i_U(t,a)da + \int_\tau^n \big(1-\theta\xi(a)\big) i_U(t,a)da, \end{aligned}$$

b) contacts with individuals infected with the sensitive strain who are symptomatic and currently under-

going treatment/prophylaxis, or will be clinically identified and given treatment:

$$Q_P(t) = \theta\Big(\delta_P \int_0^\tau \delta_s i_P(t,a)da + \delta_P \int_\tau^n i_P(t,a)da + \delta_T \int_\tau^n i_T(t,a)da$$
$$+\delta_s \int_0^\tau \xi(a) i_U(t,a)da + \int_\tau^n \xi(a) i_U(t,a)\Big),$$

c) contacts with individuals infected with the resistant strain who are asymptomatic, or those who are symptomatic and not diagnosed/treated):

$$Q_r(t) = \delta_r\Big(\delta_A A_r(t) + \delta_A A_{P,r}(t) + \delta_U I_{U,r}(t) + \delta_U I_{T,r}(t) + \delta_U I_{P,r} + \delta_s \int_0^\tau \big(1-\theta\xi(a)\big) i_{U,r}(t,a)da$$
$$+\int_\tau^n \big(1-\theta\xi(a)\big) i_{U,r}(t,a)da + (1-\theta)\int_\tau^n i_{T,r}(t,a)da$$
$$+(1-\theta)\int_0^\tau \delta_s i_{P,r}(t,a)da + (1-\theta)\int_\tau^n i_{P,r}(t,a)da\Big),$$

d) contacts with individuals infected with the resistant strain who are symptomatic and currently undergoing treatment/prophylaxis, or will be clinically identified and given treatment:

$$Q_{P,r}(t) = \theta\delta_r\Big(\int_0^\tau \delta_s i_{P,r}(t,a)da + \int_\tau^n i_{P,r}(t,a)da$$
$$+\delta_s \int_0^\tau \xi(a) i_{U,r}(t,a)da + \int_\tau^n \xi(a) i_{U,r}(t,a) + \int_\tau^n i_{T,r}(t,a)da\Big),$$

where δ_A, δ_s, δ_U, are the relative transmissibility of asymptomatic, pre-symptomatic, and stage one symptomatic infection, respectively; δ_P and δ_T represent the relative transmissibility of symptomatic infection with prophylaxis and treatment, respectively; and δ_r is the relative transmissibility of the resistant strain. It is worth noting that in the absence of prophylaxis, the model reduces to the one discussed in our previous study with only treatment of clinical infections (Alexander *et al.*, 2007b).

2 Reproduction numbers

A key descriptor in determining whether a disease can successfully establish itself and cause an outbreak in the population is the *basic reproduction number*, defined as the number of secondary infections produced by a single infected case introduced into an entirely susceptible population (Diekmann & Heesterbeek, 2000). Of particular interest is then to evaluate the impact of intervention strategies on controlling the spread of disease if implemented at an early stage of the outbreak. This evaluation is based on a quantity, the so-called control reproduction number (R_c), which reduces to the basic reproduction number in the absence of intervention measures.

To calculate R_c for the model developed here, we follow the infectious path of an infected individual in asymptomatic, pre-symptomatic, and primary and secondary stages of symptomatic infection. We first consider the introduction of an individual exposed to the sensitive strain such that $E(0) = 1$, $E(t) = 0$ for $t \in [-n,0)$, $E_P(t) = E_r(t) = E_{P,r}(t) = 0$ for $t \in [-n,0]$, and $A(0) = A_r(0) = A_P(0) = A_{P,r} = I_U(0) = I_T(0) = I_{U,r}(0) = I_{T,r}(0) = i_P(0) = I_P(0) = i_{P,r}(0) = I_{P,r}(0) = 0$. With probability $1-p$, the exposed person may undergo asymptomatic infection for the entire course of disease, and therefore generate $\beta S_0(1-p)\delta_A/\mu_A$ new cases that are not traced for post-exposure prophylaxis. An exposed individual may develop clinical

symptoms with probability p, and produce new infections during symptomatic infection without treatment, and therefore the number of new cases not encountered for prophylaxis is given by

$$R_{NN} = \beta S_0 \Big(\frac{(1-p)\delta_A}{\mu_A} + \frac{pq\delta_U}{\mu_U + d_U} + p\delta_s \int_0^\tau q(a)\big(1-\xi(a)\big)da + p\int_\tau^n q(a)\big(1-\xi(a)\big)da \Big). \tag{25}$$

Contacts of clinically infected individuals who receive treatment within the window of opportunity may be traced and given prophylaxis. The number of new cases generated through such contacts before development of drug-resistance is given by

$$R_{NP} = p\beta S_0 \Big(\frac{(1-q-V)\delta_U\delta_T}{\mu_T + d_T + \alpha} + \delta_s \int_0^\tau q(a)\xi(a)da + \int_\tau^n q(a)\xi(a)da + \delta_T \int_\tau^n \big(1-q(a)-V(a)\big)da \Big), \tag{26}$$

Introduction of an individual exposed to the sensitive strain with prophylaxis ($E_P(0)=1$), will result in the number of new infections with sensitive virus that receive no prophylaxis $R_{PN} = \delta_P \beta S_0 (1-p_P)\delta_A/\mu_{A,P}$, or may be traced and given prophylaxis

$$R_{PP} = p_P \delta_P \beta S_0 \Big(\delta_s \frac{1-e^{-\rho_P \tau}}{\rho_P} + \frac{e^{-\rho_P \tau} - e^{-\rho_P n}}{\rho_P} + \frac{\delta_U}{\mu_T + d_T + \alpha_P} e^{-\rho_P n} \Big). \tag{27}$$

We now assume that an individual exposed to the resistant strain is introduced into the population such that $E_r(0) = 1$, $E_r(t) = 0$ for $t \in [-n, 0)$, $E(t) = E_P(t) = E_{P,r}(t) = 0$ for $t \in [-n, 0]$, and $A(0) = A_r(0) = A_P(0) = A_{P,r} = I_U(0) = I_T(0) = I_{U,r}(0) = I_{T,r}(0) = i_P(0) = I_P(0) = i_{P,r}(0) = I_{P,r}(0) = 0$. Similar to the above, the number of secondary resistant infections without receiving prophylaxis is given by

$$R_{rNN} = \delta_r \beta S_0 \Big(\frac{(1-p)\delta_A}{\mu_A} + \frac{pq\delta_U}{\mu_U + d_{U,r}} + p\delta_s \int_0^\tau q(a)\big(1-\xi(a)\big)da + p\int_\tau^n q(a)\big(1-\xi(a)\big)da \Big), \tag{28}$$

and with prophylaxis, by

$$R_{rNP} = \delta_r p\beta S_0 \Big(\frac{(1-q)\delta_U}{\mu_U + d_{U,r}} + \delta_s \int_0^\tau q(a)\xi(a)da + \int_\tau^n q(a)\xi(a)da + \int_\tau^n \big(1-q(a)\big)da \Big). \tag{29}$$

Finally, we consider the introduction of an individual exposed to the resistant strain with prophylaxis into the population ($E_{P,r}(0) = 1$). Thus, the number of new infections that receive no prophylaxis is given by $R_{rPN} = \delta_r \beta S_0 (1-p)\delta_A/\mu_A$, and secondary cases who are traced and given prophylaxis is expressed as

$$R_{rPP} = p\delta_r \beta S_0 \Big(\delta_s \tau + (n-\tau) + \frac{\delta_U}{\mu_U + d_{U,r}} \Big). \tag{30}$$

Considering the next generation matrix

$$G = \begin{bmatrix} R_{NN} & R_{NP} & * & * \\ R_{PN} & R_{PP} & * & * \\ 0 & 0 & R_{rNN} & R_{rNP} \\ 0 & 0 & R_{rPN} & R_{rPP} \end{bmatrix}, \tag{31}$$

the control reproduction number is defined as the dominant eigenvalue of G (Diekmann & Heesterbeek,

2000), given by $R_c = \max\{R_1, R_2\}$, where

$$R_1 = \frac{R_{NN} + R_{PP}}{2} + \sqrt{\left(\frac{R_{NN} - R_{PP}}{2}\right)^2 + R_{NP}R_{PN}}, \tag{32}$$

$$R_2 = \frac{R_{rNN} + R_{rPP}}{2} + \sqrt{\left(\frac{R_{rNN} - R_{rPP}}{2}\right)^2 + R_{rNP}R_{rPN}}. \tag{33}$$

In the absence of antiviral therapy, assuming that the outbreak is triggered by the introduction of the drug sensitive virus, R_c reduces the basic reproduction number

$$R_0^s = \beta S_0 \left(\frac{(1-p)\delta_A}{\mu_A} + p\delta_s\tau + p(n-\tau) + \frac{p\delta_U}{\mu_U + d_U}\right). \tag{34}$$

3 Treatment rate

Effective antiviral therapy can substantially reduce the infectiousness level and the period of viral shedding in a clinical case (Jefferson *et al.*, 2006). Such effectiveness depends greatly on the initiation of treatment as early as possible within 48 hours of appearance of clinical symptoms. However, due to likely delay in seeking healthcare and therefore identification of close contacts of clinically diagnosed cases, timely application of antiviral drugs may not be achievable. This delay can significantly imperil the impact of an antiviral strategy on controlling the spread of disease in the population.

In order to evaluate the combined effect of treatment and prophylaxis, we prescribed a treatment rate $r(a)$ that decreases linearly towards the end of the window of opportunity from a maximum amount at time $a_0 \in [\tau, n]$, where a_0 represents the delay in initiating treatment after the onset of clinical symptoms. This rate corresponds to a strategy that gives priority to those who are clinically diagnosed early in this window. It is assumed that those who have not started antiviral treatment within the window of opportunity will progress to the secondary stage of symptomatic infection without receiving treatment. These assumptions give rise to the expression

$$r(a) = \begin{cases} 0 & \text{if } u < a_0 \\ b(n-a) & \text{if } a_0 \le a \le n \end{cases}$$

where b is the slope of the treatment rate $r(a)$. Letting c denote the fraction of infected cases who receive treatment so that $q(n) = 1 - c$, and using equation (11), we find $b = r_{\max}/(n - a_0)$, where $r_{\max}$ is given by

$$r_{\max} = -\frac{2\ln(1-c)}{n-\tau}.$$

Thus, the functional form of $q(a)$ with delay a_0 can be expressed as

$$q(a) = \begin{cases} 1 & \text{if } \tau \le a < a_0 \\ e^{-\frac{r_{\max}(n-a_0)}{2}} e^{\frac{r_{\max}(n-a)^2}{2(n-a_0)}} & \text{if } a_0 \le a \le n \end{cases} \tag{35}$$

4 Sensitivity Analyses

To investigate the effect of parameter changes on the results presented by simulations in the main text using baseline values given in Table 1, we performed sensitivity analyses by considering a sampling approach that allows for the simultaneous variations of several key parameters, including the relative transmissibility

(transmission fitness) of the resistant strain δ_r; the probability of developing clinical disease in the presence/absence of prophylaxis; and rates of de novo resistant mutations. Using the Latin Hypercube Sampling technique McKay *et al.* (1979), we generated samples of size $n = 100$ in which each parameter is treated as a random variable and assigned a probability function. In this technique, the parameters are uniformly distributed and sampled within their respective ranges. The range of parameter values used for sensitivity analyses are given in the main text, and the results are presented and discussed.

Table 1: Description of the model parameters with their estimated values from the published literature (Ferguson *et al.*, 2005, 2006, 2003; Gani *et al.*, 2005; Jefferson *et al.*, 2006; Longini *et al.*, 2004, 2005; Mills *et al.*, 2004; Regoes & Bonhoeffer, 2006; Stilianakis *et al.*, 1998).

Parameter	Description	Value
β	baseline transmission rate of infection	variable (day people)$^{-1}$
$1/\mu_E$	mean latent period	1.25 day
$1/\mu_A$	mean infectious period of asymptomatic infection	4.1 days
$1/\mu_U$	mean infectious period of untreated symptomatic infection (secondary stage)	2.85 days
$1/\mu_T$	mean infectious period of treated symptomatic infection (secondary stage)	1.6 days
$1/\mu_{A,P}$	mean infectious period of asymptomatic infection with prophylaxis	4.1 days
τ	mean infectious period of pre-symptomatic infection	0.25 day
n	size of the window of opportunity for start of treatment	2 days
d_U	death rate of untreated symptomatic infection	0.002 day^{-1}
d_T	death rate of treated symptomatic infection	0.0002 day^{-1}
$d_{U,r}$	death rate of symptomatic infection with the resistant strain	0.0004 day^{-1}
δ_S	relative infectiousness of pre-symptomatic infection	0.286
δ_A	relative infectiousness of asymptomatic infection	0.071
δ_U	relative infectiousness of untreated symptomatic infection (secondary stage)	0.143
δ_T	relative infectiousness of treated symptomatic infection	0.4
δ_P	relative infectiousness of symptomatic/asymptomatic infection with prophylaxis	0.2
p	probability of developing clinical symptoms without prophylaxis	0.6
p_P	probability of developing clinical symptoms with prophylaxis	0.3
ρ	rate of drug-resistance during primary symptomatic infection	0.018 day^{-1}
α	rate of drug-resistance emergence during secondary symptomatic infection	0.036 day^{-1}
ρ_P	rate of drug-resistance emergence during primary symptomatic infection with prophylaxis	0.0036 day^{-1}
α_P	rate of drug-resistance emergence during secondary symptomatic infection with prophylaxis	0.0072 day^{-1}
$1-q$	fraction of infected individuals which is diagnosed and treated	variable
V	fraction of treated individuals which develops drug-resistance within the window of opportunity	variable
ξ	probability of receiving prophylaxis following contact with an infectious case	variable

References

Alexander, M.E., Moghadas, S.M., Röst, G., Wu, J. 2008, A delay differential model for pandemic influenza with antiviral treatment, Bull. Math. Biol. 70, 382-397.

Alexander M.E., Bowman C.S., Feng Z., Gardam M., Moghadas S.M., Röst G,, Wu J,, Yan P. 2007, Emergence of drug-resistance: implications for antiviral control of pandemic influenza, Proc. R. Soc. B. 274, 1675-1684.

Diekmann, O., Heesterbeek, J.A.P. 2000, Mathematical Epidemiology of Infectious Diseases, Wiley, Chichester.

Ferguson, N.M., Cummings, D.A.T, Cauchemez, S., Fraser, C., Riley, S., Meeyai, A., Iamsirithaworn, S., Burke, D.S. 2005, Strategies for containing an emerging influenza pandemic in Southeast Asia, Nature 437, 209-214.

Ferguson, N.M., Cummings, D.A., Fraser, C., Cajka, J.C., Cooley, P.C., Burke, D.S. 2006, Strategies for mitigating an influenza pandemic, Nature 442, 448-452

Ferguson, N.M., Mallett, S., Jackson, H., Roberts, N., Ward, P. 2003, A population-dynamic model for evaluating the potential spread of drug-resistant influenza virus infections during community-based use of antivirals, J. Antimicrob. Chemother. 51, 977-990.

Gani, R., Hughes, H., Fleming, D., Griffin, T., Medlock, J., Leach, S. 2005, Potential impact of antiviral drug use during influenza pandemic, Emerg. Infect. Dis. 9, 1355-1362.

Jefferson, T., Demicheli, V., Rivetti, D., Jones, M., Di Pietrantonj, C., Rivetti, A. 2006, Antivirals for influenza in healthy adults: systematic review, Lancet 367, 303-313.

Longini, Jr., I.M., Halloran, M.E., Nizam, A., Yang, Y. 2004, Containing pandemic influenza with antiviral agents, Am. J. Epidemiol. 159, 623-633.

Longini Jr., I.M., Nizam, A., Xu, S., Ungchusak, K., Hanshaoworakul, W., Cummings, D.A.T., Halloran, M.E. 2005, Containing pandemic influenza at the source, Science 309, 1083-1087.

McKay, M., Conover, W., Beckman, R. (1979). A comparison of three methods for selecting values of input variables in the analysis of output from a computer code, *Technometrics 21*, 239-245.

Mills, C.E., Robins, J.M., Lipsitch. M. 2004, Transmissibility of 1918 pandemic influenza, Nature 432, 904-906.

Regoes, R.R., Bonhoeffer, S. 2006, Emergence of drug-resistance influenza virus: population dynamical considerations, Science 312, 389-391.

Stilianakis, N.I., Perelson, A.S., Hayden, F.G., Emergence of drug resistance during an influenza epidemic: insights from a mathematical model, J. Infect. Dis. 177, 863-873.
